# Radiomic phenotyping of the lung parenchyma in a lung cancer screening cohort

**DOI:** 10.1038/s41598-023-29058-1

**Published:** 2023-02-04

**Authors:** Babak Haghighi, Hannah Horng, Peter B. Noël, Eric A. Cohen, Lauren Pantalone, Anil Vachani, Katharine A. Rendle, Jocelyn Wainwright, Chelsea Saia, Russel T. Shinohara, Eduardo Mortani Barbosa, Despina Kontos

**Affiliations:** Department of Radiology, Perelman School of Medicine and Hospital of the University of Pennsylvania, University of Pennsylvania, 3400 Spruce Street, Philadelphia, PA 19104 USA

**Keywords:** Lung cancer, Biomarkers

## Abstract

High-throughput extraction of radiomic features from low-dose CT scans can characterize the heterogeneity of the lung parenchyma and potentially aid in identifying subpopulations that may have higher risk of lung diseases, such as COPD, and lung cancer due to inflammation or obstruction of the airways. We aim to determine the feasibility of a lung radiomics phenotyping approach in a lung cancer screening cohort, while quantifying the effect of different CT reconstruction algorithms on phenotype robustness. We identified low-dose CT scans (n = 308) acquired with Siemens Healthineers scanners from patients who completed low-dose CT within our lung cancer screening program between 2015 and 2018 and had two different sets of image reconstructions kernel available (i.e., medium (I30f.), sharp (I50f.)) for the same acquisition. Following segmentation of the lung field, a total of 26 radiomic features were extracted from the entire 3D lung-field using a previously validated fully-automated lattice-based software pipeline, adapted for low-dose CT scans. The lattice in-house software was used to extract features including gray-level histogram, co-occurrence, and run-length descriptors. The lattice approach uses non-overlapping windows for traversing along pixels of images and calculates different features. Each feature was averaged for each scan within a range of lattice window sizes (*W*) of 4, 8 and 20 mm. The extracted imaging features from both datasets were harmonized to correct for differences in image acquisition parameters. Subsequently, unsupervised hierarchical clustering was applied on the extracted features to identify distinct phenotypic patterns of the lung parenchyma, where consensus clustering was used to identify the optimal number of clusters (K = 2). Differences between phenotypes for demographic and clinical covariates including sex, age, BMI, pack-years of smoking, Lung-RADS and cancer diagnosis were assessed for each phenotype cluster, and then compared across clusters for the two different CT reconstruction algorithms using the cluster entanglement metric, where a lower entanglement coefficient corresponds to good cluster alignment. Furthermore, an independent set of low-dose CT scans (n = 88) from patients with available pulmonary function data on lung obstruction were analyzed using the identified optimal clusters to assess associations to lung obstruction and validate the lung phenotyping paradigm. Heatmaps generated by radiomic features identified two distinct lung parenchymal phenotype patterns across different feature extraction window sizes, for both reconstruction algorithms (*P* < 0.05 with K = 2). Associations of radiomic-based clusters with clinical covariates showed significant differences for BMI and pack-years of smoking (*P* < 0.05) for both reconstruction kernels. Radiomic phenotype patterns were more similar across the two reconstructed kernels, when smaller window sizes (*W* = 4 and 8 mm) were used for radiomic feature extraction, as deemed by their entanglement coefficient. Validation of clustering approaches using cluster mapping for the independent sample with lung obstruction also showed two statistically significant phenotypes (*P* < 0.05) with significant difference for BMI and smoking pack-years. Radiomic analysis can be used to characterize lung parenchymal phenotypes from low-dose CT scans, which appear reproducible for different reconstruction kernels. Further work should seek to evaluate the effect of additional CT acquisition parameters and validate these phenotypes in characterizing lung cancer screening populations, to potentially better stratify disease patterns and cancer risk.

## Introduction

Quantitative CT (QCT) imaging-based metrics, including radiomic features, can be an important tool for phenotyping lung diseases, such as COPD and interstitial lung diseases^1^, and potentially also assessing the risk for developing lung cancer^2^. For example, Raghu et al.^3^ proposed an improved model for early prediction of lung cancer from clinical, demographic and low-dose CT (LDCT) data within a lung cancer screening cohort. In addition, Hawkins et al.^4^ showed that radiomics of lung cancer screening LDCT at baseline can be used to assess risk of development of cancer. Castaldi et al.^5^ identified four subgroups of smokers within the COPDGene cohort with unique clinical characteristics and COPD-associated genetic variants. Recently, Haghighi et al.^6^ used a QCT imaging-based clustering approach to identify homogeneous clusters within current smokers with unique clinical phenotype characteristics.

The breadth of a radiomics-based approach could offer unique advantages in characterizing the heterogeneity of the lung parenchyma as an imaging biomarker of disease severity and/or the risk of developing lung cancer. Image acquisition and reconstruction can vary widely across different scanners, causing unwanted variation in extracted radiomic features^7^, which can be a challenge in standardizing and translating such imaging biomarkers. Shafiq-ul-Hassan et al.^7^ investigated the reconstruction kernel-induced variability using noise power spectra as a correction factor to reduce variability in CT texture features. Zhao et al.^8^ assess a comprehensive, commonly-used set of radiomic features from lung cancer patients and show that radiomic features can be reproducible over a wide range of imaging parameters, but smooth and sharp reconstruction algorithms can induce variability in radiomic features. Meyer et al.^9^ have also shown that most radiomic features are highly affected by CT acquisition and reconstruction, resulting in non-reproducible features in liver lesions.

Screening studies have been previously used to establish a predictive score for assessing lung diseases^10^. In this study we aim to establish the feasibility of a radiomics approach for characterizing intrinsic lung parenchymal patterns as potential surrogates of early signs of lung disease or other types of lung inflammation, which may predispose to an increased risk for lung cancer. Our main hypotheses are that lung cancer screening LDCT contain enough latent structural and functional information such that a set of comprehensive radiomic features can assess the intrinsic heterogeneity of the lung parenchyma, and that these phenotypes can be inherently robust to common CT acquisition parameters, such as reconstruction kernels. Our long-term hypothesis is that these radiomic phenotypes can serve as precursors of lung diseases as well as to characterize the extent of such diseases and to identify patients at higher risk of developing lung cancer.

## Methods

### Human data

The multicenter National Cancer Institute (NCI) Population-based Research to Optimize the Screening Process (PROSPR) lung cancer screening consortium^11^ aims to address disparities in lung cancer mortality through research on the receipt and effectiveness of lung cancer screening within and across diverse healthcare systems and patient populations. Our study was designed as a single-institution feasibility study within the NCI PROSPR-Lung consortium. This study design was approved by the institutional review board (IRB) of the University of Pennsylvania. Patient data was fully anonymized and adequate precautions were undertaken to ensure protection of patient privacy and confidentiality.

### CT acquisition parameters

We obtained LDCT scans (n = 308) acquired with Siemens Healthineers scanners from patients undergoing routine lung cancer screening at our institution between 2015–2018, that had two different sets of image reconstruction kernels available (i.e., medium (I30f.), sharp (I50f.)) for the same acquisition (*Two-kernel data set*). LDCT images had the lowest slice thickness of 1 mm. Within the same institutional lung cancer screening cohort, we also identified an independent sample of patients screened who also had Pulmonary Function Test (PFT) data and COPD obstruction information available in their clinical record (n = 88) (*PFT data set*). Additional available clinical covariates for all patients included in our study were age, BMI, sex, Lung-RADs^12^, smoking status, smoking pack-years and cancer diagnosis (i.e., biopsy confirmed cancer cases). Lung-RADs categories were collapsed into two groups based on scan findings: Group A: negative scan (Lung-RADS 1 and 2) and Group B: positive scan (Lung-RADs 3/4A/4B/4X). The demographics and clinical information of the two independent data sets are summarized in Tables 1 and 2, respectively.

**Table 1:** Demographics of study sample with two LDCT reconstruction kernels by SIEMENS scanners. Numerical data presented as Means (standard deviations).

Demographics of study sample	
	N = 308
Age	64.8 (5.84)
BMI	27.08 (6.04)
Smoking pack-years	52.47 (24.85)
Sex (female/male)	147/161
Smoking status (current/former)	158/150
Lung-RADs (A/B)	262/46
Cancer (no/yes)	293/15

Lung-RADs categories 1and 2 are grouped as (A) and 3/4A/4B/4X as (B). Value range: Age (48–68), BMI (18–31).

**Table 2:** Demographics of study sample with lung obstruction information obtained from pulmonary function test (PFT). Numerical data presented as Means (standard deviations).

Demographics of the PFT dataset	
	N = 88
Obstruction (no/yes)	49/64
Age	64.76 (5.89)
BMI	28.04 (6.91)
Smoking pack-years	48.67 (21.45)
Sex (female/male)	52/35
Smoking status (current/former)	32/56
Lung-RADs (A/B)	73/15
Cancer (no/yes)	82/6

Lung-RADs categories 1 and 2 are grouped as (A) and 3/4A/4B/4X as (B). Value range: Age (49–71), BMI (18–32).

### Radiomic phenotyping of the lung parenchyma

**Figure 1:**
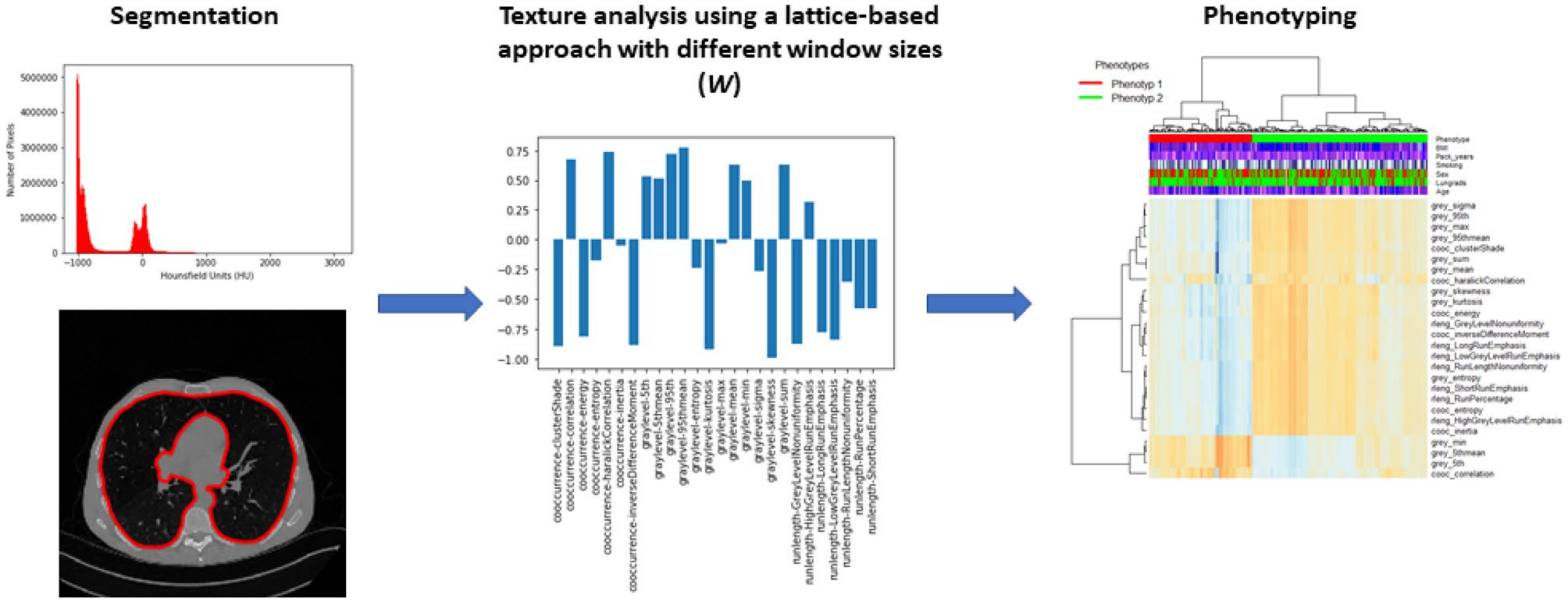
The schematic of the LDCT analysis pipeline including lung field segmentation, radiomic feature extraction, and unsupervised hierarchical clustering for phenotype generation.

**Figure 2:**
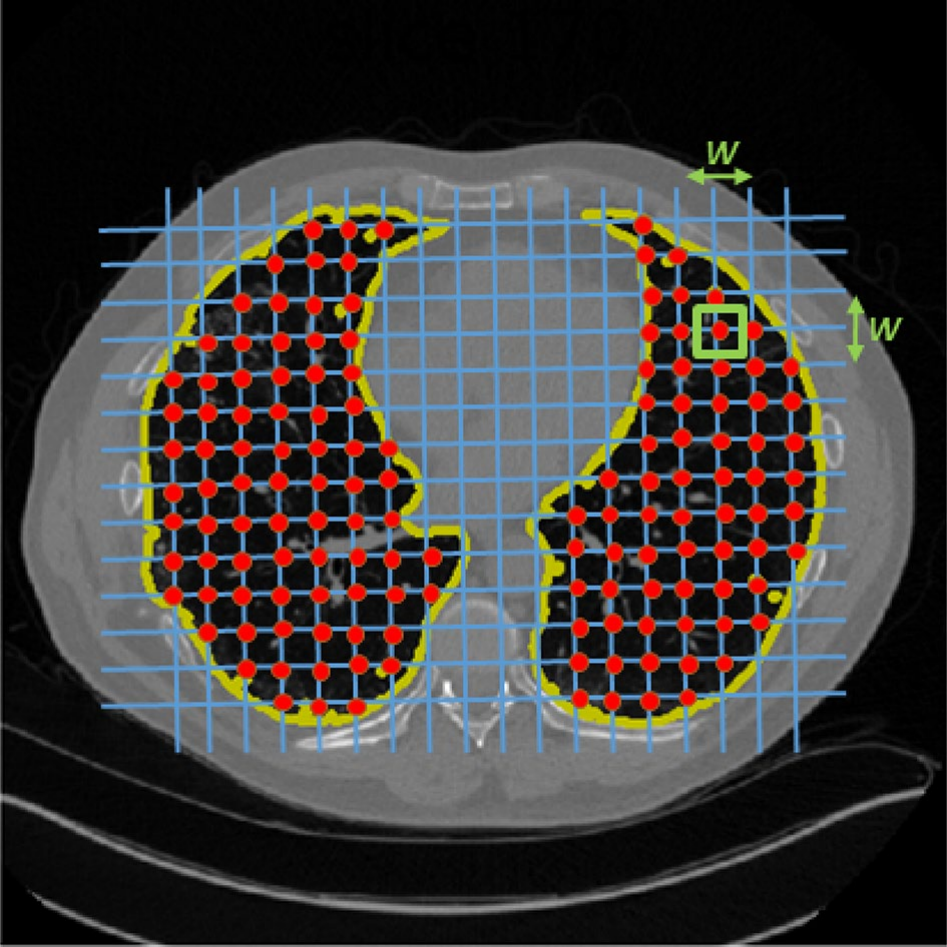
The schematic of the lattice-based feature extraction approach, with window size (*W*). This approach can extract a range of local tissue texture features obtained within a window (i.e., the green rectangle with size of *W*) surrounding each lattice point (i.e., the red intersection points on the regular grid drawn using blue lines) for characterizing the parenchymal tissue heterogeneity.

The lung field in all LDCT images for these two datasets was segmented. Our segmentation method is an automated 3-dimensional, intensity-based algorithm using *K-means* clustering to properly determine cluster centers of air / lung tissue versus soft tissue attenuation. The threshold-based segmentation excludes vessels when segmenting the lung domain and furthermore, we have clinical information about patients with nodule cases and we cross-checked this with our segmentation result. After segmentation, the lattice-based texture feature extraction pipeline^13^ was applied to extract 26 three dimensional (3D) radiomic features from three major statistical approaches, gray-level histogram, co-occurrence, and run-length descriptors. Briefly, gray-level histogram features are first-order statistics describing the distribution of gray-level intensities. Co-occurrence features consider the spatial relationships of pixel intensities in different directions and are based on the gray-level co-occurrence matrix that encodes the relative frequency of neighboring intensity values. Run-length features capture the coarseness of texture in specified directions by measuring strings of consecutive pixels that have the same gray-level intensity along specific linear orientations (please see the supplementary section for feature definitions). Different window sizes (*W*) from 4 to 20 mm were used to assess texture information at different spatial scales at each lattice point with an intent to evaluate different spatial levels of texture alterations. Furthermore, for each window size *W,* measures from lattice points were averaged over each 3D feature map to create a per-patient measure for each feature. This resulted in a *feature vector* of 26 features characterizing parenchymal complexity for each patient. The overall feature extraction pipeline is shown in Fig. 1 and the schematic of the lattice approach is depicted in Fig. 2. The pipeline is fully automated including preprocessing (anonymization and normalization), segmentation and machine learning part (clustering and statistical analysis).

### Feature harmonization

The extracted imaging feature vectors from both datasets (two-kernel and PFT) were harmonized to correct for differences in imaging parameters using ComBat^14^. ComBat is a harmonization method originally developed for genomic datasets that can address and correct variation in imaging features due to heterogeneity in imaging parameters—such as reconstruction kernel—by assuming a location and spread variation in the distribution of each feature due to the imaging parameter value, using an empirical Bayes approach to estimate these location and spread parameters, and inverse transforming the values by these estimated parameters to harmonize the data. When it is expected that other non-feature covariates will affect the feature values—for instance, if we expect the features to be associated with sex—these covariates can be specified as *protected variables* in the ComBat procedure, and variation due to these covariates will be preserved in the ComBat harmonization. Before applying ComBat, outlying texture feature values were identified, as previous work had shown that excluding outliers improved the effectiveness of ComBat harmonization. To identify outliers, in each dataset, each feature was residualized as the dependent variable in each of three univariable linear regression models, with age at index scan, BMI, and smoking pack-years as the predictors. Any image in which any of the three residuals, for any feature, was outside the range of the median ± 2.5 × IQR was tagged as an outlier. ComBat was used to harmonize features from the datasets both with and without the outliers dropped, using the Python *neurocomBat* package, with kernel as the batch effect for the two-kernel dataset and manufacturer as the batch effect for the PFT dataset. Sex, Lung-RADS score, smoking status, age, BMI, and smoking pack-years were protected variables in the ComBat harmonization.

### Clustering and statistical analysis

With the extracted feature vectors for each patient, and for each window size *W*, an unsupervised hierarchical clustering approach was applied to the feature vectors extracted from each scan, and separately for each of the two reconstruction kernels, to group patients that share similar lunch parenchymal patterns. Therefore, the clusters of patients were derived for each reconstruction kernel. Consensus clustering was used to find the optimal number of clusters for each reconstruction kernel^15^. Entanglement parameters^16^ showing the quality of the alignment between different trees of hierarchical clustering from the two kernels were computed (Fig. S2). Entanglement is a measure between 1 (full entanglement) and 0 (no entanglement), where a lower entanglement coefficient corresponds to a better alignment between the clustering dendrogram structures.

### Phenotype associations with demographics and PFT data

We evaluated associations between the identified radiomic lung parenchymal phenotype clusters with the available demographic and clinical covariates. The Kruskal–Wallis and chi-square tests were used to assess differences from continuous and categorical variables, respectively, across phenotypes where *P* value = 0.05 was used as the threshold for determining significance in all tests. All data analysis was performed using the software R (version 3.1.1).

To assess the degree of reproducibility (validation) of cluster characteristics from one data set to another, the derived clusters in one data set (i.e., the two-reconstruction kernel dataset) were mapped to another data set domain (i.e., the independent PFT dataset). First, the centroids of two clusters from *the Kernel data set* were calculated. Then, the mapped clusters were assigned in *the PFT data set* by assigning each patient to the closest cluster centroid learned by the hierarchical clustering algorithm in the Kernel data set (please see supplementary section for details).

### Ethics approval and consent to participate

Ethics were approved by the IRB and the PROSPR steering committee. Waiver of consent was approved by the IRB. We confirm that all methods were carried out in accordance with relevant guidelines and regulations.

### Informed consent

This was a retrospective study. Waiver of Informed consent was obtained by our IRB for all subjects and/or their legal guardian(s).

## Results

### Feature harmonization

Differences in feature distributions between kernel groups in the raw features and ComBat-harmonized features were assessed with the Kolmogorov–Smirnov (KS) test at a *P* value significance level of 0.05. KS testing on the feature distributions of the two-reconstruction kernel dataset prior to harmonization demonstrated statistically significant differences between features for kernel groups with different window sizes. Also the number of features with statistically significant differences decreased when residual outliers were dropped after harmonizing by ComBat (Table S1).

Similarly, the PFT dataset features showed statistically significant differences between manufacturer groups prior to harmonization for different window sizes. After applying ComBat and outlier dropping, the number of features with statistically significant differences decreased (Table S2).

### Two clusters and imaging-based characteristics

A consensus clustering approach was applied to the harmonized imaging feature vectors, and the number of clusters K = 2 with a significant difference (*P* < 0.05) was selected as the optimal number based on their consensus matrices, which was consistent across the different window sizes (*W*) used for feature extraction (see supplementary materials for details). The optimal number of clusters was calculated using consensus clustering (Fig. S1).

**Figure 3:**
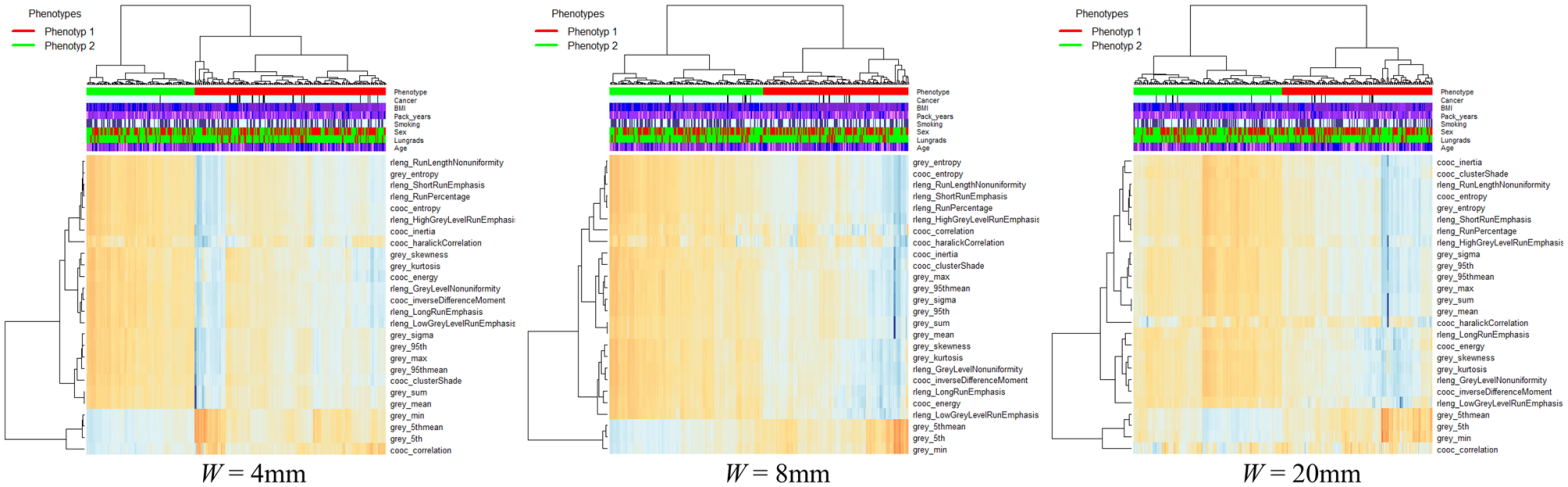
Heatmaps generated by unsupervised hierarchical clustering from extracted radiomic features for different window sizes (*W*) and for the I130f reconstruction kernel (medium kernel). Each column represents a subject/LDCT scan and each row a specific radiomic feature. The dendrogram at the top represents the grouping of patients in distinct phenotypes, whereas the dendrogram on the left represents groupings of extracted features based on their similarity. Associations with the clinical covariates are shown on the top legends.

**Figure 4:**
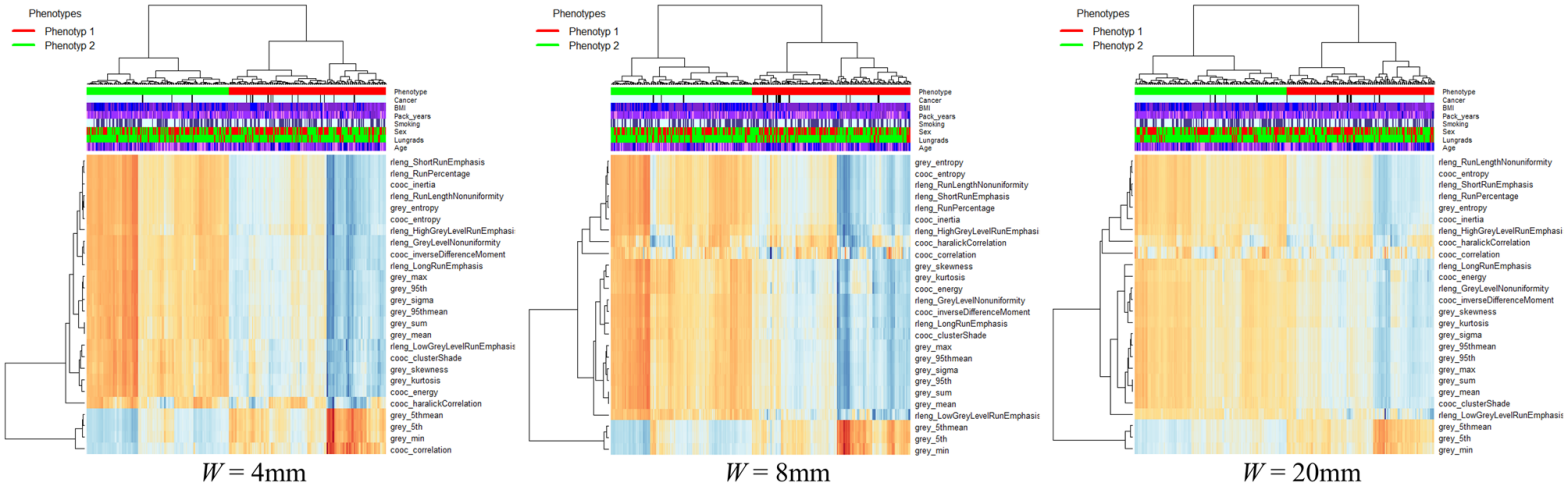
Heat map generated by unsupervised hierarchical clustering of extracted radiomic features for I150f (medium kernel) reconstruction parameters for phenotypic patterns (red and blue colors) for different window sizes (*W*). Each column in heat map represents a patient and each row represents a specific radiomic feature. Dendrogram at top represents grouping of patients in distinct phenotypes, whereas dendrogram on left represents groupings of extracted features with similar information. Associations with the clinical covariates are shown on the top legends.

Heatmaps generated by radiomic features identified with two distinct parenchymal phenotype patterns across different window sizes for both reconstruction algorithms are depicted in Figs. 3 and 4, consistently showing two statistically significant phenotypes across both reconstruction kernels and all window sizes (*P* < 0.05). The number of patients differed slightly between kernels and windows sizes. The clustering results are tabulated in Table 3.

**Table 3:** Associations of clinical covariates for the two reconstruction kernels with their corresponding imaging phenotype clusters for different window sizes *W* = 4, 8 and 20 mm after feature harmonization. Numerical data presented as Means (standard deviations).

	I130f. Kernel		*P value*	I150f. Kernel		*P* value
	Cluster 1	Cluster 2		Cluster 1	Cluster 2	
*Clinical covariates (window size* = *4 mm)*						
	N = 197	N = 111		N = 162	N = 146	
Age	64.96 (5.78)	64.25 (5.79)	0.34	65.19 (5.8)	64.16 (5.74)	0.12
BMI	25.63 (5.33)	29.6 (6.52)	< 0.0001	25.03 (5.15)	29.31 (6.26)	< 0.0001
Sex (female/male)	92/105	55/56	0.72	76/86	71/75	0.85
Smoking status (current/former)	105/92	53/58	0.41	85/77	73/73	0.75
Smoking pack-years	54.04 (25.81)	50.21 (23.75)	0.2	55.32 (27.29)	49.71 (22.19)	0.05
Lung-RADs (A/B)	165/32	97/14	0.49	135/27	127/19	0.46
Cancer (no/yes)	183/14	110/1	0.031	150/12	143/3	0.050

	I130f. Kernel		*P* value	I150f. Kernel		*P* value
	Cluster 1	Cluster 2		Cluster 1	Cluster 2	
*Clinical covariates (window size* = *8 mm)*						
	N = 146	N = 154		N = 159	N = 141	
Age	65.19 (5.92)	64.21 (5.62)	0.143	65.31 (5.84)	63.99 (5.65)	0.045
BMI	24.83 (4.57)	29.26 (6.59)	< 0.0001	24.89 (5.02)	29.6 (6.27)	< 0.0001
Sex (female/male)	64/82	79/75	0.24	73/86	70/71	0.60
Smoking status (current/former)	75/71	77/77	0.90	82/77	70/71	0.83
Smoking pack-years	55.78 (27.75)	50.36 (22.60)	0.064	55.64 (27.62)	50.02 (22.22)	0.055
Lung-RADs (A/B)	126/20	131/23	0.89	134/25	123/18	0.57
Cancer (no/yes)	139/8	147/7	0.25	148/11	138/4	0.091

	I130f. Kernel		*P* value	I150f. Kernel		*P* value
	Cluster 1	Cluster 2		Cluster 1	Cluster 2	
*Clinical covariates (window size* = *20 mm)*						
	N = 157	N = 154		N = 152	N = 156	
Age	65.43 (5.89)	64.01 (5.58)	0.029	65.14 (6.06)	64.28 (5.48)	0.19
BMI	24.97 (4.67)	29.22 (6.56)	< 0.0001	24.88 (5.17)	29.18 (6.17)	< 0.0001
Sex (female/male)	69/88	78/76	0.28	72/80	75/81	0.99
Smoking status (current/former)	80/77	78/76	0.98	81/71	77/79	0.56
Smoking pack-years	55.8 (27.52)	49.55 (22.26)	0.029	55.08 (26.34)	50.3 (23.71)	0.095
Lung-RADs (A/B)	133/24	132/22	0.93	126/26	136/20	0.37
Cancer (no/yes)	148/9	148/6	0.62	141/11	152/4	0.101

Entanglement parameters assessing the degree of similarity between clustering dendrograms were calculated to be 0.26, 0.16, 0.9 for *W* = 4, 8, and 20 mm, respectively (Fig. S2). The smaller entanglement parameters indicate that the two clusters for the two different kernels are more similar to each other when using *W* = 4 and 8 mm as compared to 20 mm. Furthermore, the mapped PFT dataset also produced two statistically significant clusters (*P* < 0.05) for different kernels and window sizes (Table 4).

**Table 4:** Associations of clinical covariates for PFT data with their corresponding clusters for window sizes *W* = 4, 8 and 20 mm after feature harmonization and cluster mapping. Numerical data presented as Means (standard deviations).

	Data with PFTs		*P* value
	Cluster 1	Cluster 2	
*Clinical covariates (window size* = *4 mm)*			
	N = 45	N = 43	
Age	65.13 (5.05)	64.7 (6.43)	0.73
BMI	25.56 (5.8)	30.51 (7.45)	0.001
Smoking pack-years	46.56 (18.61)	55.79 (24.45)	0.049
Obstruction (no/yes)	20/25	18/25	0.43
Sex (female/male)	23/22	29/14	0.18
Smoking status (current/former)	21/24	11/32	0.07
Lung-RADs (A/B)	34/11	39/4	0.11
Cancer (no/yes)	44/2	38/4	0.59

	Data with PFTs		*P* value
	Cluster 1	Cluster 2	
*Clinical covariates (window size* = *8 mm)*			
	N = 45	N = 43	
Age	65.53 (5.37)	63.54 (6.55)	0.11
BMI	25.36 (5.4)	31.6 (7.22)	< 0.0001
Smoking pack-years	48.33 (20.2)	49.42 (23.76)	0.05
Obstruction (no/yes)	22/23	16/27	0.42
Sex (female/male)	34/11	18/25	0.18
Smoking status (current/former)	16/31	17/26	0.07
Lung-RADs) (A/B)	42/3	32/11	0.1
Cancer (no/yes)	43/2	39/4	0.6

	Data with PFTs		*P* value
	Cluster 1	Cluster 2	
*Clinical covariates (window size* = *20 mm)*			
	N = 46	N = 42	
Age	64.33 (5.88)	65.54 (6.09)	0.375
BMI	30.24 (6.34)	22.91 (5.43)	< 0.0001
Smoking pack-years	48.51 (23.21)	48.62 (18.39)	0.053
Obstruction (no/yes)	23/24	16/25	0.52
Sex (female/male)	36/11	17/24	0.84
Smoking status (current/former)	17/30	15/26	0.08
Lung-RADs) (A/B)	42/5	31/10	0.2
Cancer (no/yes)	44/3	36/5	0.7

### Clusters associations with demographic and PFT data

Association between clusters (phenotypes) for the two reconstruction kernels with demographics is tabulated in Table 3 for the different lattice window sizes (*W* = 4, 8 and 20 mm). BMI showed a significant difference between clusters (*P* < 0.05), consistently across all window sizes. Furthermore, smoking pack-years showed significant levels for *W* = 8 and 20 mm. Cancer diagnosis showed a significant difference for two clusters for smaller window sizes *W* = 4 and 8 mm while this difference was not significant for *W* = 20 mm (*P* > 0.05). Lung-RADs did not show any significant difference across the two phenotype clusters for any window size.

To assess reproducibility of the clustering approaches between the two datasets, the mapped clusters from *the two-kernel data* set to *the PFT data set* were assessed. Table 4 shows the associations between the clinical covariates for PFT dataset and their corresponding mapped clusters for different window sizes. The similar clustering approaches for the PFT data also showed two statistically significant phenotypes (*P* < 0.05). Association with the available PFT and clinical covariates for the different window sizes is shown in Table 4. While airway obstruction did not show significant difference, BMI and smoking pack-years demonstrated significant differences across clusters for all window sizes.

## Discussion

Lung diseases, such as history of emphysema, chronic bronchitis, pneumonia and tuberculosis, are shown to influence lung cancer risk, independently of tobacco use^17^. One of the related hypotheses is that such diseases, which obstruct the airflow in the lung airways, are sources of inflammation in the lung tissue and may act as a catalyst in the development of lung neoplasms. Clinically established assessment exists for evaluating the extent of such diseases, including PFT, which evaluates degree of pulmonary impairment for example after respiratory infections, chronic bronchitis and can assess the severity of emphysema and COPD. However, most of these diseases are shown to be heterogeneous, both across and within patients, and such measures may have limitations in capturing the extent of the inflammation and obstruction on the lung tissue, likely associated with differential cancer risk^18^. LDCT offers a unique opportunity to characterize the heterogeneity of lung parenchyma as a potential surrogate of such diseases conferring increased lung cancer risk using refined quantitative imaging measures^19^. Thus, the main premise of our study is that radiomic imaging features can aid in characterizing phenotypes of lung parenchymal heterogeneity from LDCT, and that these phenotypes are relatively robust to image acquisition. Ultimately, these phenotypes may relate to underlying biologic heterogeneity of the overall lung structure, potentially related to lung inflammation or early disease manifestation, that may increase lung cancer risk.

We applied unsupervised hierarchical clustering to a comprehensive set of LDCT radiomic features to establish feasibility of deriving intrinsic lung parenchymal phenotypes in a lung screening cohort, and further evaluated their reproducibility across different reconstruction kernels and feature extraction parameters such as window size. Furthermore, we applied our approach to an independent dataset with PFT information to assess the ability of our phenotypic approach in distinguishing patients with the same imaging and clinical characteristics. We found that these phenotypes were relatively robust across settings. This can be helpful when dealing with heterogeneous CT image data from different acquisition parameters.

Cluster phenotype assignments were dependent on variation in LDCT reconstruction kernels and feature extraction parameters. The degree of similarity between clusters was evaluated using the entanglement parameter and the window sizes with the smaller entanglement were considered as the optimal region of interest sizes in radiomic feature extraction process for decreasing phenotype sensitivity to variation of LDCT parameters. The phenotypes were also found to be reproducible in an independent dataset. For this study, window sizes of *W* = 4 mm and 8 mm showed the lowest sensitivity to CT reconstruction parameters. This implies that the window size parameter showing the degree of information that can be extracted from images at different spatial scales is important in evaluating different levels of lung texture alterations. The lower entanglement parameters, along with the fact that a smaller window size can potentially allow for extracting more refined image texture information, suggests that smaller window sizes (*W* = 4 mm and 8 mm) can be the optimal for radiomic feature extraction and the corresponding phenotypes.

The two phenotypes across the different kernel and windows settings showed significant differences related with BMI. This could be because higher BMI (obesity) can contribute to higher levels of systemic inflammation, diabetes, and worse prognosis in many infectious conditions, as suggested by Sood et al.^20^ and Joppa et al.^21^, which can in turn affect our detected cluster characteristics. Interestingly, and considering that lung tissue inflammation may be a risk factor for the development of lung cancer, our results also showed a significant difference for cancer diagnosis between the two clusters, which was a consistent observation across kernels. These two clusters also had significantly different smoking pack-years for *W* = 4 mm which is another well-established risk factor for lung cancer diagnosis^22^. Thus, together this data suggests that radiomic phenotypes may represent intrinsic lung parenchymal characteristics that may reflect underlying biological underpinnings of the lung tissue predisposition to lung cancer, and may ultimately have value in augmenting risk assessment. Better identification of patients at high risk from lung cancer continues to be very important when prioritizing the best candidates for inclusion in lung cancer screening programs.

When applying our phenotyping approach using cluster mapping on the independent screening LDCT data set with PFT information, the degree of lung obstruction as measured by PFT did not show statistically significant differences, suggesting that radiomic phenotyping may capture complementary information to the current gold standard. However, smoking pack-years showed significant differences across the two phenotypes for smaller window sizes (*W* = 4 mm and 8 mm). Nevertheless, cancer diagnosis and Lung-RADs did not reach significance, which may be due to the relatively small number of cancer cases in this lung screening cohort.

Our study has several limitations. First, our work focused on mainly evaluating the effect of LDCT reconstruction kernel parameters on the extracted phenotypes, which is only one factor of the LDCT acquisition. Future analyses should also consider additional LDCT parameters such as dose, image resolution, and slice thickness (Fig. S4)^23^. Also, different important factors such as lung size or diffusing capacity of the lungs for carbon monoxide (DLCO) can be considered for better assessment of correlation with clinical information. Second, for better assessment of phenotype stability over time, our analysis can be extended to available longitudinal data. Third, our study included a relatively small sample confirmed cancer cases, therefore future larger studies are needed, with additional clinical information such as history of pulmonary/vascular conditions, asthma, emphysema, and shortness of breath, and more extensive lung obstruction data, including the Fleischner Society emphysema grading system^24^ in order to expand these analyses in more heterogeneous LDCT datasets and further evaluate potential associations between such possible phenotypes, lung diseases, and the risk for developing lung cancer.

In conclusion, our study demonstrated the feasibility of leveraging a radiomics-based approach to identify potentially intrinsic phenotypes of lung parenchymal patterns in LDCT screening scans. We showed that such phenotypes are reproducible in an independent dataset, and are relatively robust when considering variations in LDCT reconstruction kernel and the resolution/scale of the radiomics feature extraction approach. We also demonstrated a significant association with these phenotypes and BMI and cancer diagnosis, which could represent a phenotypic manifestation of inflammation to the lung parenchymal structure.

## Supplementary Information

Supplementary Information.

## Data Availability

The datasets generated and/or analyzed during the current study are not currently publicly available but are available from the corresponding author on reasonable request.

## Code availability

Codes can be accessible upon request.
