# Supplementary material for: Radiomic phenotyping of the lung parenchyma in a lung cancer screening cohort
Source: Sci Rep. 2023 Feb 4;13:2040. doi: 10.1038/s41598-023-29058-1 (PMC9899203; doi:10.1038/s41598-023-29058-1)
Supplement: Supplementary file 1 — Supplementary Information. [file 41598_2023_29058_MOESM1_ESM.docx]

**SUPPLEMENTARY**

**Imaging features**

Lattice-based texture estimation adopted for lung 3D CT scan was used for feature extraction. Three sets of features were derived: Gray-level histogram, Co-occurrence, Run-length features.

**Gray-level histogram features** are first-order histogram statistics calculated from the gray-level intensity histogram of each image.

| *Feature* | *Mathematical Notation* | *Qualitative description* |
| --- | --- | --- |
| Mean | $\frac{\sum_{k} k*g(k)}{K}$ | Mean gray-level value |
| Min | min($k$) | Minimum gray-level value |
| Max | max($k$) | Maximum gray-level value |
| 5^th^ Percentile | $k$: 5% of values ≤ $k$ | The histogram bin that 5% of gray-level values are less than or equal to. |
| 5^th^ Mean | $\frac{\sum_{k} k*g(k)}{\sum_{k} g(k)}$ for $k$ ≤ 5^th^ Percentile | Mean value of the gray-level values which less than or equal to the 5^th^ Percentile. |
| 95^th^ Percentile | $k$: 95% of values ≥ $k$ | The histogram bin that 95% of gray-level values are less than or equal to. |
| 95^th^ Mean | $\frac{\sum_{k} k*g(k)}{\sum_{k} g(k)}$ for $k$ ≥ 95^th^ Percentile | Mean value of the gray-level values which larger than or equal to the 95^th^ Percentile. |
| Sum | $\sum_{k} k*g(k)$ | Sum of gray-level values |
| Sigma | $\sqrt{\sum_{k} \left( k-Mean \right)^{2}*g(k)}$ | Variation of gray-level values around the Mean |
| Entropy | $-\sum_{k} g\left( k \right)*log(g(k))$ | Measure of histogram uniformity |
| Kurtosis | $Sigma^{-4}\sum_{k} \left( k-Mean \right)^{4}*g(k)-3$ | Measure of histogram flatness |
| Skewness | $Sigma^{-3}\sum_{k} \left( k-Mean \right)^{3}*g(k)$ | Measure of histogram symmetry |
| where $k$ is the histogram bin, *g* is the frequency of the histogram bin and $K=\sum_{k} g(k)$. | | |

**Co-occurrence features** capture the spatial relationship between pixels and are based on the gray-level co-occurrence matrix (GLCM). In a GLCM, each element, $f(i,j)$, corresponds to the frequency with which two neighboring pixels, one with gray level $i$ and the other with $j$, occur within a specified distance ($d$). In our study, the GLCM matrices were estimated using different $window sizes=4, 6, 8, 10, 12, 14, 16, 18, 20$ pixels.

| *Feature* | *Mathematical Notation* | *Qualitative description* |
| --- | --- | --- |
| Cluster Shade | $\sum_{ij} \left( i-\mu_{i}+j-\mu_{j} \right)^{3}*f(i,j)$ | Asymmetry in gray-level values |
| Correlation | $\sum_{ij} \frac{\left( i-\mu_{i} \right)*\left( j-\mu_{j} \right)*f(i,j)}{\sigma_{i}{*\sigma}_{j}}$ | Linear gray-level dependence |
| Haralick Correlation | $\sum_{ij} \frac{ij*f\left( i,j \right)-\mu_{i}*\mu_{j}}{\sigma_{i}{*\sigma}_{j}}$ |  |
| Energy | $\sum_{ij} f\left( i,j \right)^{2}$ | Certainty of gray-level co-occurrence |
| Entropy | $-\sum_{ij} f\left( i,j \right)*log(f\left( i,j \right))$ | Uncertainty of gray-level co-occurrence |
| Inertia | $\sum_{ij} \left( i-j \right)^{2}*f(i,j)$ | Local variation of gray-level intensity |
| Inverse Difference Moment | $\sum_{ij} \frac{f(i,j)}{1+\left( i-j \right)^{2}}$ | Local homogeneity in gray-level values |
| where $\mu_{i}=\sum_{j} i*f(i,j)$, $\mu_{j}=\sum_{i} j*f(i,j)$, $\sigma_{i}^{2}=\sum_{j} \left( i-\mu_{i} \right)^{2}*f(i,j)$, and $\sigma_{j}^{2}=\sum_{i} \left( j-\mu_{j} \right)^{2}*f(i,j)$. | | |

**Run-length features** capture the coarseness of a texture in specified directions, where a run is defined as a string of consecutive pixels with similar gray-level intensity along specific linear orientation. Similar to GLCMs, a run-length matrix $R$ is defined, with each element, $R\left( i,j \right)$*,* representing the number of runs with pixels of gray-level intensity equal to $i$ and length of run equal to $j$ along the specific orientation. The size of the matrix $R$ is $M\times N$, where $N$ is equal to the maximum run length. The following run-length statistics were estimated using different $window sizes=4, 6, 8, 10, 12, 14, 16, 18, 20$ pixels.

| *Feature* | *Mathematical Notation* | *Qualitative description* |
| --- | --- | --- |
| Gray Level Non-uniformity | $\frac{1}{n_{r}}{\sum_{i=1}^{M} \left( \sum_{j=1}^{N} R\left( i,j \right) \right)}^{2}$ | Dissimilarity in runs across gray-level values |
| High Gray Level Run Emphasis | $\frac{1}{n_{r}}\sum_{i=1}^{M} \sum_{j=1}^{N} R\left( i,j \right)*i^{2}$ | Runs of high-gray-level values |
| Long Run Emphasis | $\frac{1}{n_{r}}\sum_{i=1}^{M} \sum_{j=1}^{N} R\left( i,j \right)*j^{2}$ | Emphasis on the long runs |
| Low Gray Level Run Emphasis | $\frac{1}{n_{r}}\sum_{i=1}^{M} \sum_{j=1}^{N} \frac{R\left( i,j \right)}{i^{2}}$ | Runs of low-gray-level values |
| Run Length Non-uniformity | $\frac{1}{n_{r}}{\sum_{j=1}^{N} \left( \sum_{i=1}^{M} R\left( i,j \right) \right)}^{2}$ | Dissimilarity in runs across lengths |
| Run Percentage | $\frac{n_{r}}{\#pixels}$ | Homogeneity and distribution of runs |
| Short Run Emphasis | $\frac{1}{n_{r}}\sum_{i=1}^{M} \sum_{j=1}^{N} \frac{R\left( i,j \right)}{j^{2}}$ | Emphasis on the short runs |
| where $n_{r}=\sum_{i=1}^{M} \sum_{j=1}^{N} R\left( i,j \right)$ is the total number of runs. | | |

**Clustering Analysis**

| 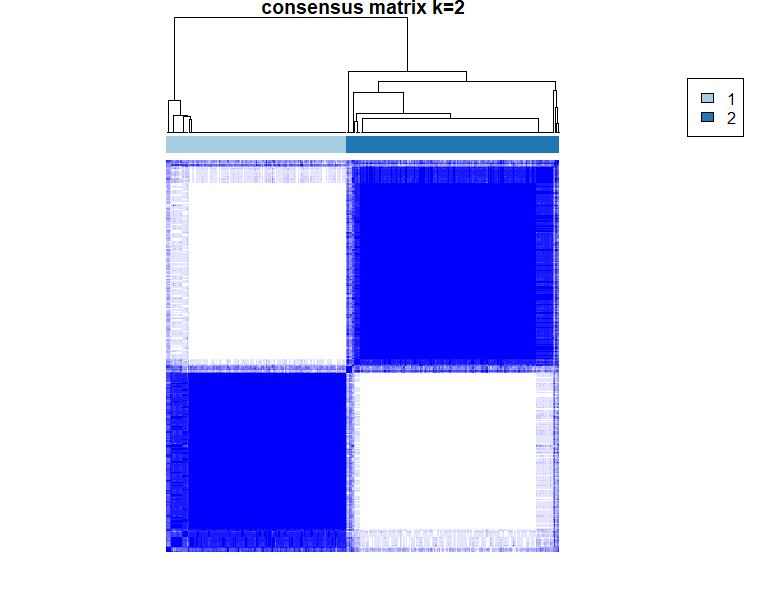 | 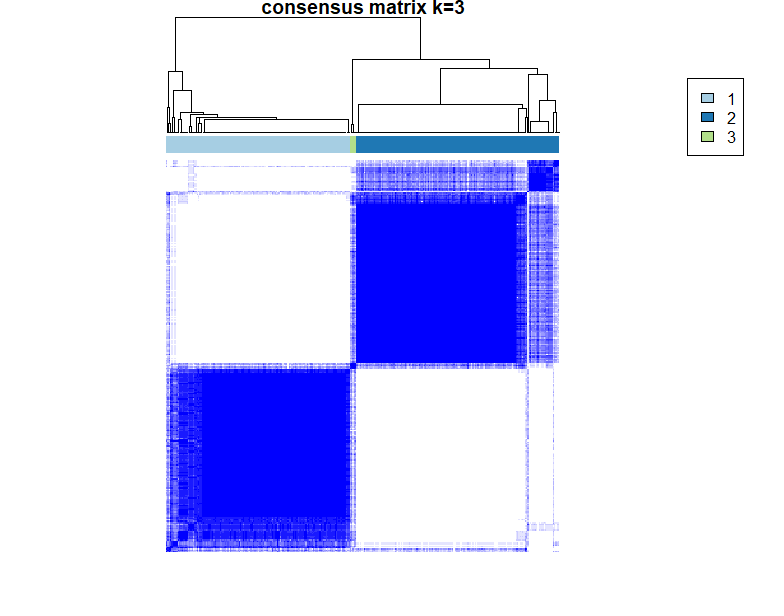 | | 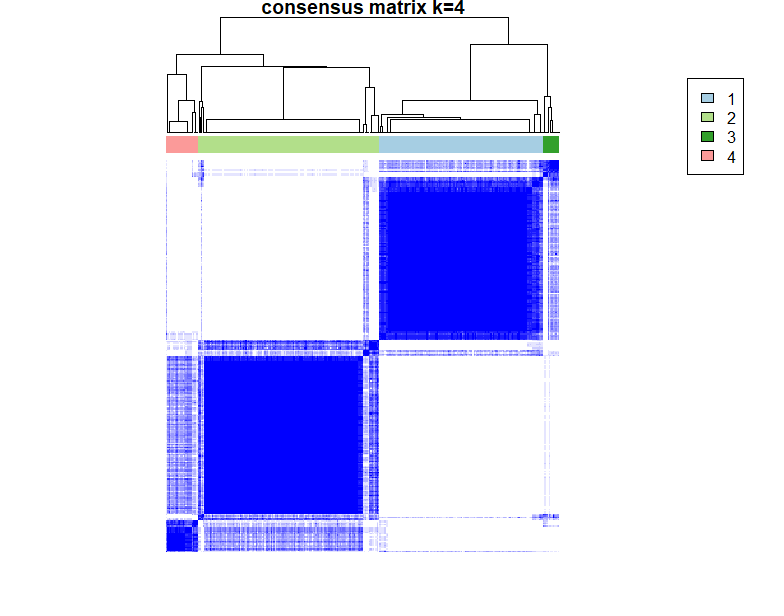 |
| --- | --- | --- | --- |
| *W* = 4mm | | | |
| 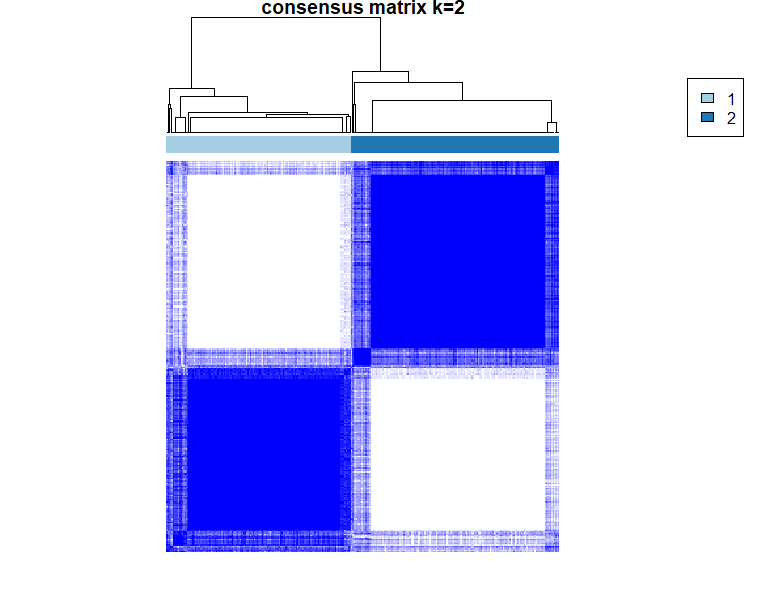 | 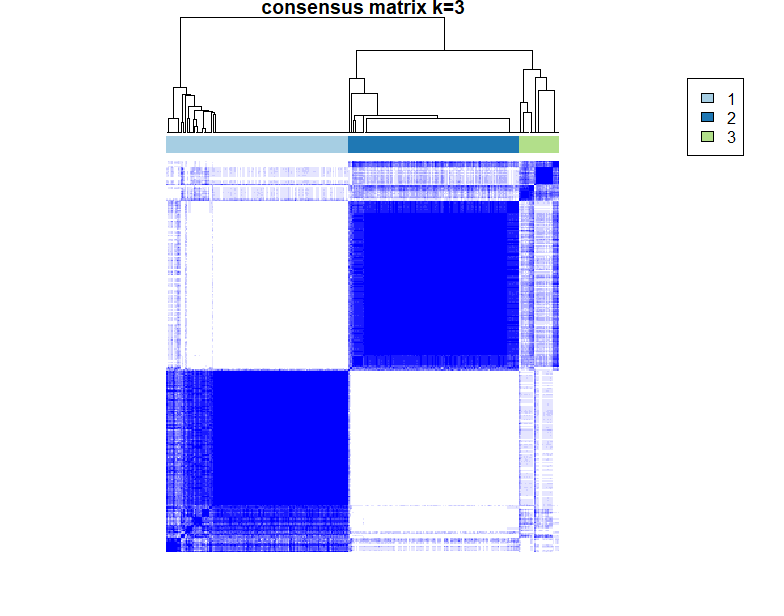 | 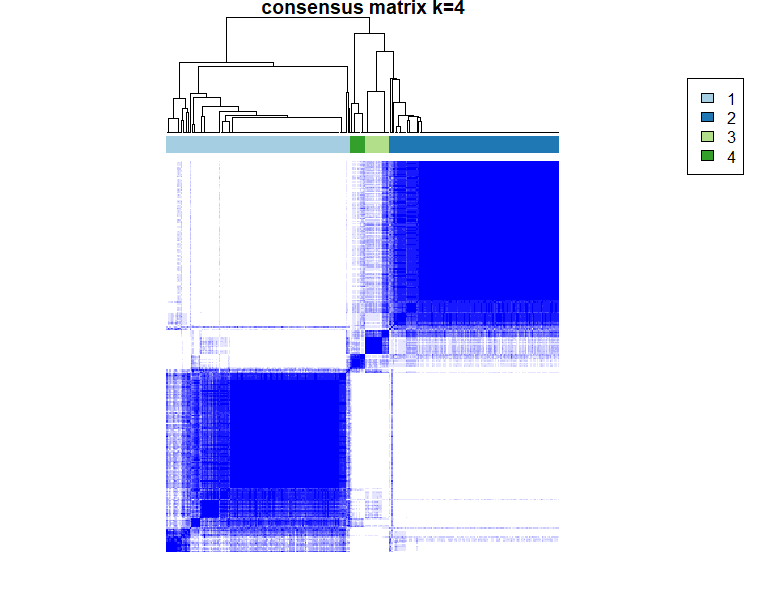 | |
| *W* = 8mm | | | |
| 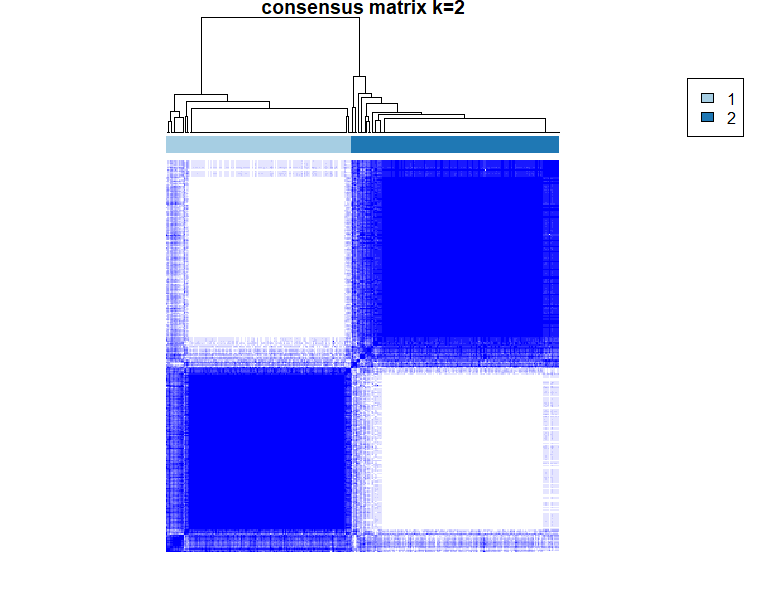 | 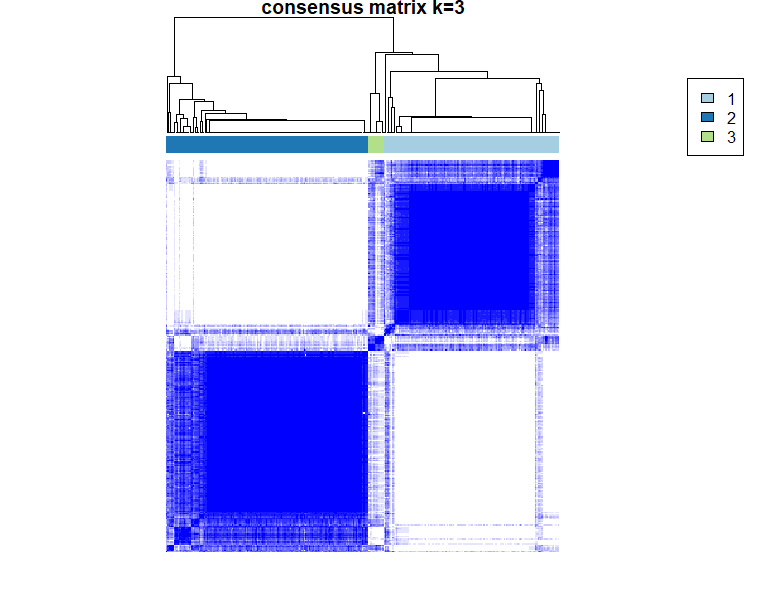 | 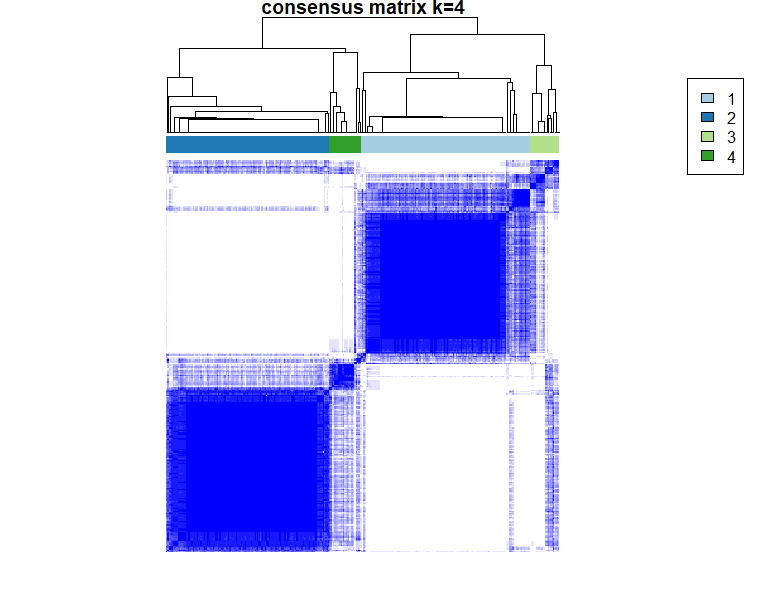 | |
| *W* = 20mm | | | |
| **Fig. S1:** Consensus clustering for different number of clusters (K = 2, 3, 4) for finding the best optimal cluster for patients with I130f kernel. | | | |

**Entanglement**

| 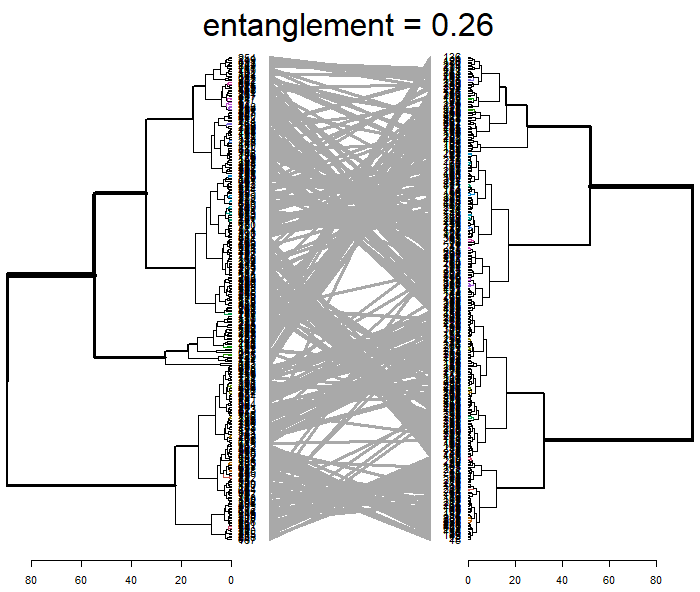 | 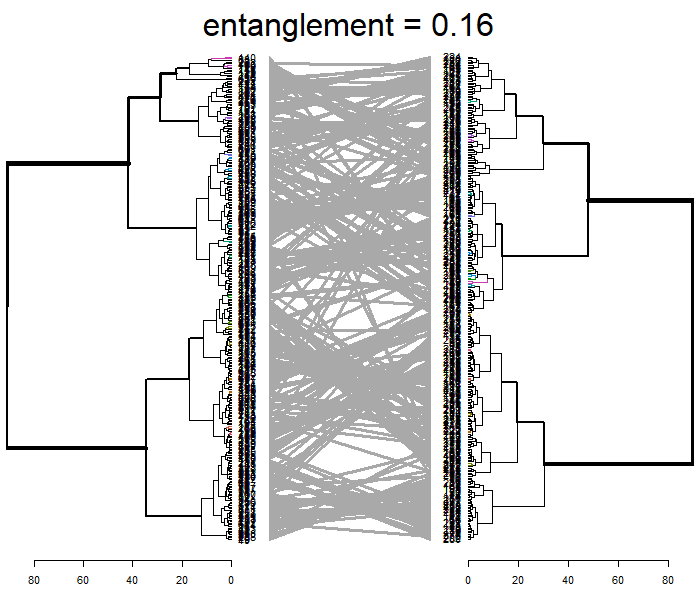 | 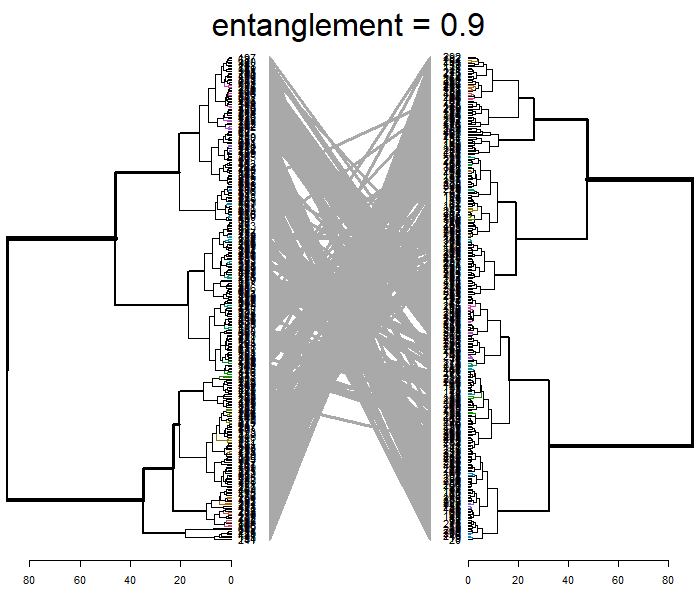 |
| --- | --- | --- |
| *W* = 4mm | *W* = 8mm | *W* = 20mm |
| **Fig. S2:** Entanglement after applying feature harmonization comparing the two clusters between reconstruction kernels. | | |

**Feature Harmonization**

Outlier detection in the harmonization process resulted in 8 patients (images) being classified as outliers from the *W* = 4mm, 8mm, and 20mm window sizes in the two-reconstruction kernel dataset. Similarly, 12, 15, and 36 patients were detected as outliers from the *W* = 4mm, 8mm, and 20mm in the PFT dataset. The effects of dropping scans with residuals classified as outliers are shown for a sample representative feature (gray entropy) and covariate (BMI) from the two-reconstruction kernel dataset in Figure S3. The outlier removal resulted in a more even distribution of imaging features.


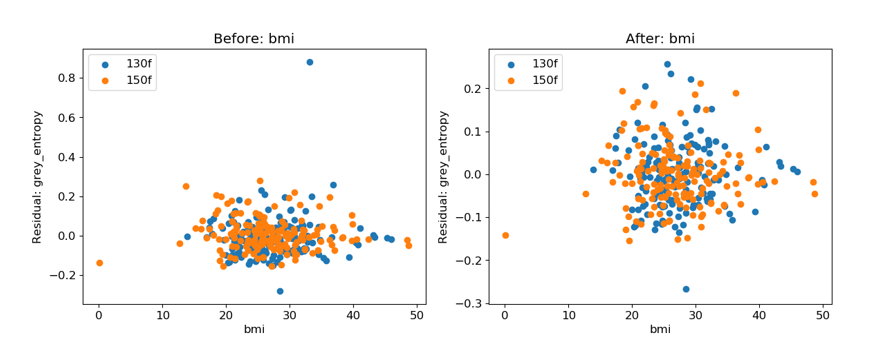


**Figure S3**. Residual plots for gray entropy versus BMI for the raw features (left) and the ComBat-harmonized features with the outliers dropped (right) for the two-reconstruction kernel dataset with window size 4mm.

**Table S1.** Number of features with statistically significant differences in distribution due to reconstruction kernel as detected with the KS test (*p* < 0.05) for the two-reconstruction kernel dataset.


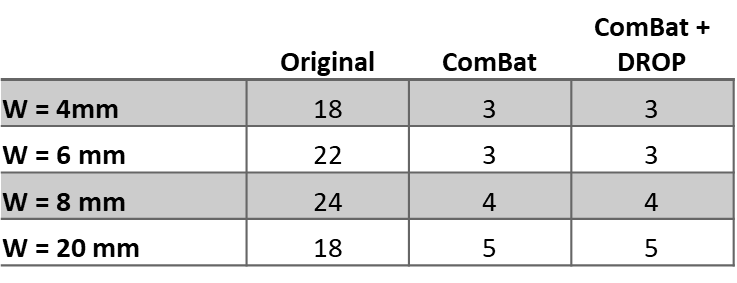


**Table S2.** Number of features with statistically significant differences in distribution due to the reconstruction kernel as detected with the KS test (*p* < 0.05) for the PFT dataset.


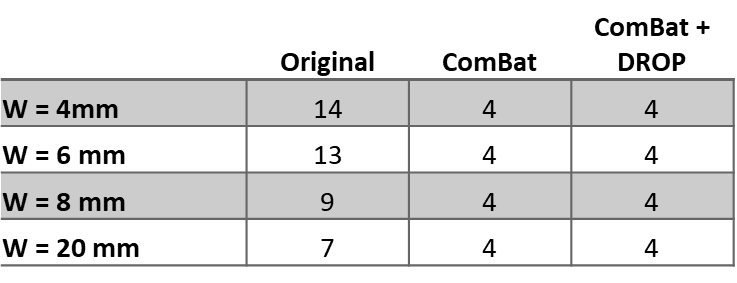


**CT Acquisition Parameters**

Images used in study have voxel size of 0.5mm x 0.5mm x 0.5mm. Some CT parameters are shown in Figure S4.

| 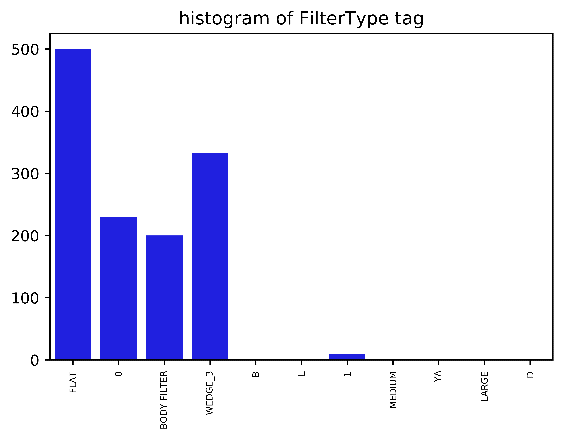 | 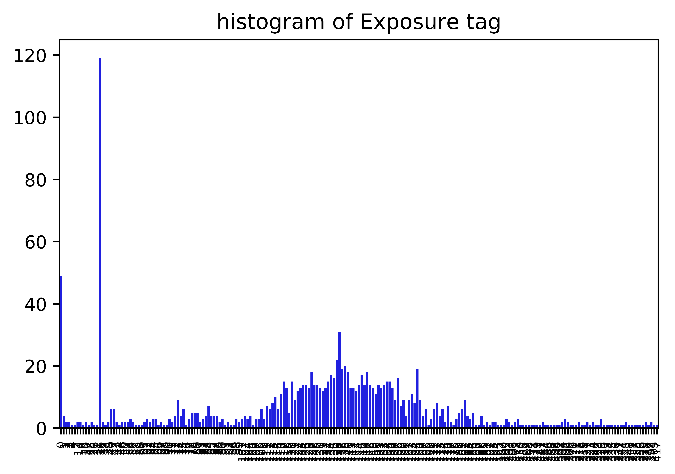 |
| --- | --- |
| 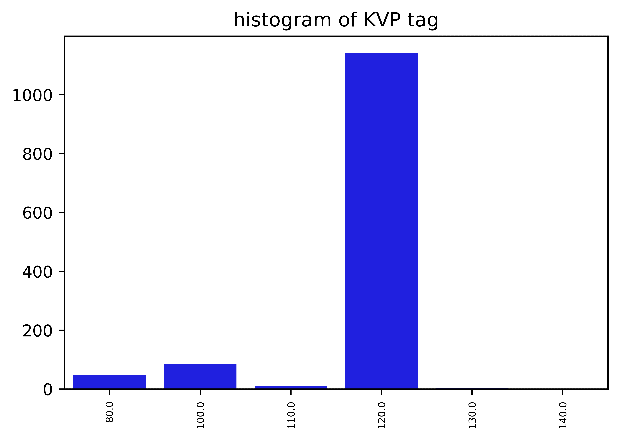 | 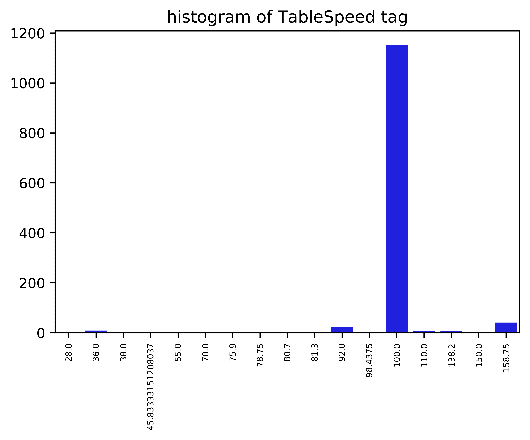 |
|  |  |
| **Fig. S4**. Some CT acquisition parameters used for image analysis | |

## Availability of Data and Material

The datasets generated and/or analyzed during the current study are not publicly available due to privacy and its proprietary nature but are available from the corresponding author on reasonable request.
